# Effects of Hexane on Protein Profile, Solubility and Foaming Properties of Defatted Proteins Extracted from *Tenebrio molitor* Larvae

**DOI:** 10.3390/molecules26020351

**Published:** 2021-01-12

**Authors:** Alexia Gravel, Alice Marciniak, Manon Couture, Alain Doyen

**Affiliations:** 1 Department of Food Sciences, Institute of Nutrition and Functional Foods (INAF), Université Laval, Quebec City, QC G1V 0A6, Canada; alexia.gravel.1@ulaval.ca (A.G.); alice.marciniak.1@ulaval.ca (A.M.); 2 Department of Biochemistry, Microbiology and Bio-Informatics, Institute of Integrative Biology and Systems (IBIS), Université Laval, Quebec City, QC G1V 0A6, Canada; manon.couture@bcm.ulaval.ca

**Keywords:** *Tenebrio molitor*, hexane defatting step, protein concentrates, protein profile and identification, protein solubility, foaming capacity

## Abstract

Inclusion of edible insects in human diets is increasingly promoted as a sustainable source of proteins with high nutritional value. While consumer acceptability remains the main challenge to their integration into Western food culture, the use of edible insects as meal and protein concentrate could decrease neophobia. The defatting of edible insects, mostly done with hexane, is the first step in producing protein ingredients. However, its impact on protein profiles and techno-functionality is still unclear. Consequently, this study compares the protein profiles of hexane-defatted and non-hexane-defatted yellow mealworm (*Tenebrio molitor*) meals and protein extracts, and evaluates the impact of hexane on protein solubility and foaming properties. Results showed that profiles for major proteins were similar between hexane-defatted and non-defatted samples, however some specific content differences (e.g., hexamerin 2) were observed and characterized using proteomic tools. Protein solubility was markedly lower for *T. molitor* meals compared to protein extracts. A large increase in the foaming capacity was observed for defatted fractions, whereas foam stability decreased similarly in all fractions. Consequently, although the hexane-defatting step was largely studied to produce edible insect protein ingredients, it is necessary to precisely understand its impact on their techno-functional properties for the development of food formulations.

## 1. Introduction

As food security challenges related to worldwide population growth are anticipated in the coming years, an interest in insects as an emerging protein source for human consumption is being promoted. More specifically, Western countries have shown great interest in yellow mealworm (*Tenebrio molitor*) due to its high protein content and composition of essential lipids, as well as the low environmental impact of farming insects [1]. Nevertheless, food neophobia is the major challenge, which negatively affects the social acceptability of this alternative food resource [2,3]. It was suggested that the integration of edible insects as meal and protein concentrates or isolates in different food formulations could enhance consumer acceptability [2,3,4,5]. Consequently, numerous studies have investigated the nutritional and functional properties (e.g., solubility, foaming, gelling and emulsions) [6,7,8,9] of insect protein powders as a function of processing methods, mainly blanching and drying, in order to optimize the ingredient quality [10,11].

Defatting of raw food materials and by-products using organic solvents is a frequent method for producing protein-enriched ingredients [12,13,14]. Hexane is the most frequently used solvent for the production of defatted insect meal and protein extracts [15,16,17] despite its environmental, economic and safety disadvantages [18]. These drawbacks have motivated the study of different extraction solvents (ethanol, methanol, etc.) [19,20], as well as more sustainable options (supercritical CO_2_ lipid extraction) [19,21]. Nevertheless, hexane remains the most popular method to remove lipid from the solid insect matrix for its high efficiency and availability [18]. Until recently, very few studies were available describing the effects of solvent and defatting parameters on the composition of protein extracts and their techno-functional properties. Kim et al. (2020) evaluated the impact of different organic solvents on the functional properties of defatted proteins extracted from *Protaetia brevitarsis* larvae. These authors determined that hexane-defatted protein fractions had better amino acid composition, protein solubility and functional properties compared to the same fractions defatted with methanol and ethanol [20]. Borremans et al. (2020) compared the functional properties of full-fat and hexane-defatted *T. molitor* meals and demonstrated that the defatted meal’s foaming capacity was equivalent to that of egg albumen, indicating its potential as alternative foaming agents in food formulation [22]. However, the detailed effects of hexane-defatting on the protein profile of *T. molitor* meals and protein extracts, their solubility and foaming properties were never evaluated. Consequently, the aims of this study were to compare and determine the impact of hexane-defatting on the proximate composition of *T. molitor* meal and protein extract fractions. Specific emphasis was given to the modification of protein profiles by proteomic tools. Protein solubility and foaming properties of defatted and non-defatted meals and protein extracts were determined.

## 2. Materials and Methods

### 2.1. Raw Materials

Living mealworm larvae (*T. molitor*) at age of 73 days were kindly provided by Neoxis (Saint-Flavien, Québec, Canada). First, mealworm larvae were separated from feed substrate and frass residues by passing through a coarse filter. Next, larvae were killed by freezing at −30 °C without starvation. Frozen mealworm larvae were freeze-dried, ground with a household compact blender (Magic Bullet, Los Angeles, CA, USA) and passed through an 800 µm mesh sieve to produce *T. molitor* meal (TMI). This meal was stored at −20 °C before further analysis and processing. All experiments were performed in triplicate using three separate insect batches.

### 2.2. Methods

#### 2.2.1. Lipid Extraction

Half of the TMI meal from each insect batch was stored at −20 °C while the other half was defatted using the Soxhlet method as described by Tzompa-Sosa et al. (2014) [23]. Briefly, 10 g of TMI were weighed into a cellulose cartridge (Fisher Scientific catalog number 12-101-100) and the lipids were extracted with hexane for 6 h. The defatted *T. molitor* meal (TMD) was recovered, vacuum-dried in an oven at 50 °C for 5 h and stored at −20 °C before further analysis and processing.

#### 2.2.2. Protein Extraction

Soluble proteins from the TMI and TMD fractions were recovered as described by Yi et al. (2013), with modifications [24]. First, TMI and TMD fractions were solubilized in a 9.5 mM ascorbic acid solution in a 1:8 (*w*/*v*) ratio and magnetically stirred overnight at 10 °C. The suspensions were then centrifuged at 15,000× *g* for 30 min at 4 °C and the supernatant containing the soluble proteins was filtered three times through a Whatman^®^ Grade 4 filter paper in a Büchner funnel. Both filtrates were freeze-dried, producing two different fractions: a hexane-defatted soluble protein (HDSP) and a non-hexane-defatted soluble protein (NHSP) extract from *T. molitor*.

**Figure 1:**
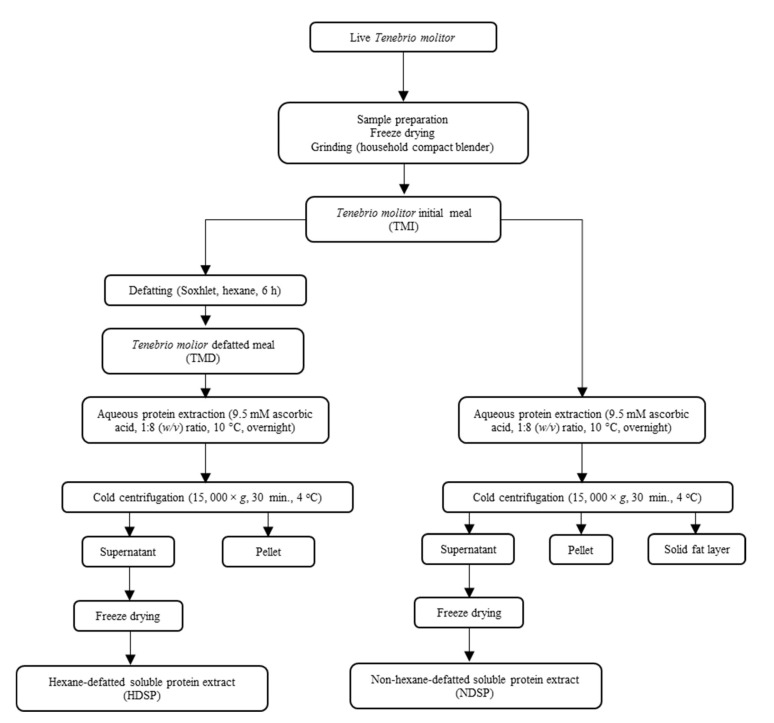
Experimental design for the production of defatted and non-defatted mealworm meals and protein extracts.

The detailed methodology used to generate the different fractions is illustrated in Figure 1.

### 2.3. Analysis

#### 2.3.1. Proximate Composition

The proximate composition was determined for the four *T. molitor* fractions (TMI, TMD, HDSP, and NDSP). The protein content was measured using the Dumas Method [19,25] (Elementar rapid Micro N cube, Langenselbold, Germany), with a nitrogen-to-protein conversion factor of 4.76 for TMI and TMD, and 5.60 for HDSP and NDSP, accounting for the high chitin content of TMI and TMD fractions and therefore allowing for a more accurate protein content determination, as suggested by Janssen et al. (2017) [26]. The lipid content was determined using the Mojonnier method (AOAC 925.32). Moisture and ash content were determined by official methods AOAC 950.46 (A) and AOAC 920.153, respectively [27]. The method described by Spinelli et al. (1974) was used to determine the chitin content of the four fractions [28].

#### 2.3.2. Protein Profile

The two-dimensional (2D) gel analysis was carried out according to Kumar et al. (2017), with modifications [29]. Briefly, 2 mg of proteins from the four *T. molitor* fractions were mixed with the sample buffer containing 7 M urea, 2 M thiourea, 4% CHAPS (*w*/*v*), 1% Triton-X-100 (*v*/*v*), 20 mM Tris, 1% DTT (*w*/*v*) and 0.5% pH 3-10 IPG buffer (*v*/*v*). A volume of 250 μL of each sample solution (TMI, TMD, HDSP and NHSP) was loaded on a 13 cm GE Healthcare Immobiline^®^ DryStrip (pH 3–10) and first-dimension isoelectric focusing (IEF) was performed using the Ettan IPGphor 3 IEF system (GE Healthcare, Piscataway, NJ, USA) at 20 °C, 50 μA/strip, 30 V for 12 h (rehydration), 100 V for 1 h, 500 V for 1 h, 1000 V for 1 h, 5000 V for 1 h and then 8000 V until 16,000 Vh was reached. The first-dimension strips were kept at −20 °C until used for the second dimension. For the second dimension, each strip was soaked in 20 mL of equilibration buffer containing 6 M urea, 2 M Milli-Q H_2_O, 50 mM Tris-HCl pH 8.8, 30% glycerol (*v*/*v*), 2% SDS (*v*/*v*), 2% DTT (*w*/*v*) and traces of bromophenol blue for 15 min at 20 °C. SDS-PAGE was carried out at 10 °C using 17.5 cm × 16.8 cm × 1 mm 15% polyacrylamide gels topped with 5% polyacrylamide stacking gels. The migration was performed at 25 μA for each gel. The running buffer consisted of 1X tris-glycine SDS solution (Bio-Rad, Hercules, CA, USA). The molecular weights (MW) of mealworm proteins were estimated by using a MW marker (Prestained Protein Standards Precision Plus Protein™ All Blue, Bio-Rad, cat. #1610373, Hercules, CA, USA). Proteins were fixed by soaking the gels in a 50% MeOH (*v*/*v*) and 10% acetic acid (*v*/*v*) solution for 12 h. Gels were then stained by soaking in GelCode™ Blue Stain Reagent (Thermo Fisher Scientific, Whaltham, MA, United-States) for 24 h, rinsed in Milli-Q water and scanned on a ChemiDoc™ MP Imaging System (Bio-Rad, Hercules, CA, USA). The 2-D gel image analysis was performed using ImageJ software according to the procedure of Natale et al. (2011), with modifications [30]. Briefly, gel images were aligned using the same image as reference with the bUnwarpJ plugin and repetitions for TMI, TMD, HDSP, and NDSP were stacked and summed to give a single gel image representative of all 3 repetitions.

#### 2.3.3. Protein Identification by Mass Spectrometry

##### Mass Spectrometry

Mass spectrometry experiments on selected 2D gel spots were performed by the Proteomics platform of the CHU de Quebec Research Center, Quebec, Canada. Samples were analyzed by nanoLC/MSMS using a Dionex UltiMate 3000 nanoRSLC chromatography system (Thermo Fisher Scientific, San Jose, CA, USA) connected to an Orbitrap Fusion mass spectrometer (Thermo Fisher Scientific, San Jose, CA, USA). Peptides were trapped at 20 μL/min in loading solvent (2% acetonitrile, 0.05% TFA) on a 5 mm × 300 μm C18 pepmap cartridge pre-column (Thermo Fisher Scientific/Dionex Softron GmbH, Germering, Germany) over 5 min. The pre-column was then switched online with a Pepmap Acclaim column (Thermo Fisher Scientific, San Jose, CA, USA)—a 50 cm × 75 µm internal diameter separation column—and the peptides were eluted with a linear gradient from 5–40% solvent B (A: 0.1% formic acid, B: 80% acetonitrile, 0.1% formic acid) over 35 min, at 300 nL/min. Mass spectra were acquired using a data dependent acquisition mode with Thermo XCalibur software version 4.1.50. Full scan mass spectra (350 to 1800 *m*/*z*) were acquired in the orbitrap using an AGC target of 4e5, a maximum injection time of 50 ms and a resolution of 120,000. Internal calibration was done using lock mass on the *m*/*z* 445.12003 siloxane ion. Each MS scan was followed by MSMS fragmentation of the most intense ions for a total cycle time of 3 s (top speed mode). The selected ions were isolated using the quadrupole analyzer in a window of 1.6 *m*/*z* and fragmented by Higher energy Collision-induced Dissociation (HCD) with 35% of collision energy. The resulting fragments were detected by the linear ion trap at rapid scan rate with an AGC target of 1e4 and a maximum injection time of 50 ms. Dynamic exclusion of previously fragmented peptides was set for a period of 20 s and a tolerance of 10 ppm.

##### Database Searching

Mascot Generic Format (MGF) peak list files were created using Proteome Discoverer 2.3 software (Thermo Fisher Scientific, San Jose, CA, USA). The MGF sample files were then analyzed using Mascot (Matrix Science, London, UK; version 2.5.1). Mascot was set up to search a contaminant database and Uniprot *Tenebrionidae* database (40,907 entries), assuming that the digestion enzyme was trypsin. Mascot was searched with a fragment ion mass tolerance of 0.60 Da and a parent ion tolerance of 10.0 ppm. Carbamidomethyl of cysteine was specified in Mascot as a fixed modification. Deamidation of asparagine and glutamine and oxidation of methionine were specified in Mascot as variable modifications. Two missed cleavages were allowed.

##### Criteria for Protein Identification

Scaffold (version Scaffold_4.8.4, Proteome Software Inc., Portland, OR, USA) was used to validate MS/MS-based peptide and protein identifications. A false discovery rate of 1% was used for peptide and protein. Proteins that contained similar peptides and could not be differentiated based on MS/MS analysis alone were grouped to satisfy the principles of parsimony. The total spectrum count value, which corresponds to the total number of spectra identified for a protein, was used to identify selected protein spots from the 2D gels, as this parameter was reported to be a semi-quantitative measure for a given protein abundance in proteomic studies [31,32].

### 2.4. Protein Solubility

Suspensions of TMI, TMD, HDSP, and NDSP fractions (1% *w/v*) were prepared in Milli-Q water. The pH of suspensions was adjusted to 5, 7 or 9 with 1 M NaOH or 1 M HCl. The suspensions were centrifuged at 20,000× *g* for 30 min at 20 °C and filtered through a Whatman^®^ Grade 1 filter paper. The filtrates were freeze-dried and the recovered fractions were analyzed for their protein content by the Dumas method with a 4.76 and 5.60 nitrogen-to-protein conversion factor, respectively for the meal larvae (TMI and TMD) and for the protein extracts (HDSP and NDSP) as described previously [26]. The protein solubility was calculated using Equation (1) [33]:

(1) $$Solubility\left(\%\right)=\frac{protein\;content\;in\;supernatant}{total\;protein\;content\;in\;sample}\times 100$$

### 2.5. Foaming Properties

Foaming capacity (*FC*) and foam stability (*FS*) were determined as described by Zielińska et al. (2018) with some modifications [7]. A volume of 30 mL of a 3% (*w*/*v*) TMI, TMD, HDSP, or NDSP sample was whipped using a hand mixer (KitchenAid, KHM512IB, Benton Charter Township, MI, USA) at a speed of approximately 800 rpm for 10 min and immediately transferred into a cylinder. The total volumes of foam were read at time 0 (*V*_0_), and then every 15 min until 90 min following (*V_t_*). Foaming capacity was calculated using Equation (2) and *FS* were calculated using Equation (3) [34]:

(2) $$FC\left(\%\right)=\frac{V_{0}-30}{30}\times 100$$

(3) $$FS\left(\%\right)=\frac{V_{t}-30}{V_{0}-30}\times 100$$

### 2.6. Statistical Analysis

The proximate composition, functional properties experiments, and 2D gels were all performed in triplicate. Proximate composition data were subjected to a one-way analysis of variance (ANOVA) and functional properties experiments data were subjected to a two-way ANOVA using the Statistical Analysis System (SAS) University Edition, SAS^®^ Studio 3.5 software. The differences of the means between the samples were determined using the Tukey test. *p*-values < 0.05 were considered statistically significant.

## 3. Results

### 3.1. Proximate Composition of Meals and Protein Extracts

Table 1 presents the proximate compositions of the four *T. molitor* fractions. The total solids contents were quite similar for all fractions (meals and protein extracts) with values ranging from 90.3 (TMI) to 95.2% (TMD). The defatting step applied to TMI meal was efficient, since very low lipid concentrations (0.3–0.4%) were obtained in TMD and HDSP. Of all meals and protein extracts, TMI had the lowest protein content with a value of 38.6%. The defatting step largely improved protein recovery in defatted fractions with respective values of 59.1% and 62.7% for TMD and HDSP samples. The defatting step also helped to improve protein content in the extracts since the protein recovery in HDSP (62.7%) was increased by 9% compared to NDSP (54.0%) (Table 1). In addition, chitin content was higher in TMD (7.8%) compared to TMI (5.0%), and after aqueous solubilization, no chitin was detected in defatted (HDSP) and non-hexane-defatted (NDSP) soluble protein extracts.

### 3.2. Profiles and Characterization of Proteins in Meals and Protein Extracts

**Figure 2:**
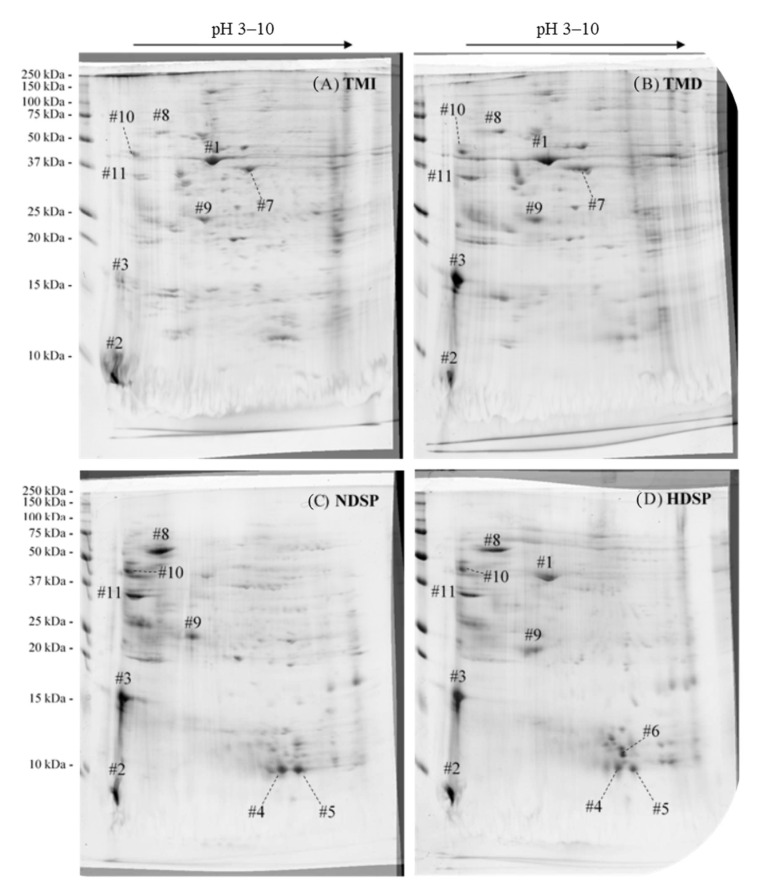
Two-dimensional electrophoresis of *T. molitor* proteins for (**A**) TMI, (**B**) TMD, (**C**) NDSP, and (**D**) HDSP. Proteins composing each numbered spot were characterized by proteomics (Table 2).

Figure 2 presents the protein profiles of TMI, TMD, HDSP, and NDSP fractions obtained after 2D SDS-PAGE analysis. The 11 main spots (#1 to #11) visualized in 2D gels were excised for protein characterization by proteomic tools (Table 2). As observed for 2D gels (Figure 2), proteomic results confirmed that *T. molior* fractions (TMI, TMD, HDSP, and NDSP) have very similar protein profiles consisting of muscular (e.g., actin-87E-like protein) and hemolymph proteins (e.g., hexamerin 2, 12 kDa hemolymph protein b) as well as proteins associated with various metabolic activities (e.g., α-amylase, chitinase, arginine kinase). However, some differences in spot intensities were observed between gels. While the cockroach allergen-like protein (spot #2) and the 28 kDa desiccation stress protein (spot #9) were present in all fractions with similar relative intensities, spot #3, identified as a cockroach allergen-like protein, was present at high intensity for each *T. molitor* fraction except for TMI. Similarly, the actin-87E-like protein (spot #1) was present in all fractions, except for NDSP where the intensity of the protein spot was low. Furthermore, 12 kDa hemolymph protein b (spot #4 and #5), α-amylase (spot #8), the melanin-inhibiting protein (spot #10), as well as chitinase (spot #11) were predominantly abundant in soluble protein extracts NDSP and HDSP. Conversely, an arginine kinase fragment (spot #7) could only be found in whole meals TMI and TMD. Finally, the 86 kDa early-staged encapsulation inducing protein and hexamerin 2 (spot #6) were only abundant in HDSP. It is worth noting that some identified proteins were associated only with other insects from the *Tenebrionidae* family, such as *Tribolium castaneum*, since they were absent from the Uniprot database for *T. molitor* (Table 2).

### 3.3. Protein Solubility

**Figure 3:**
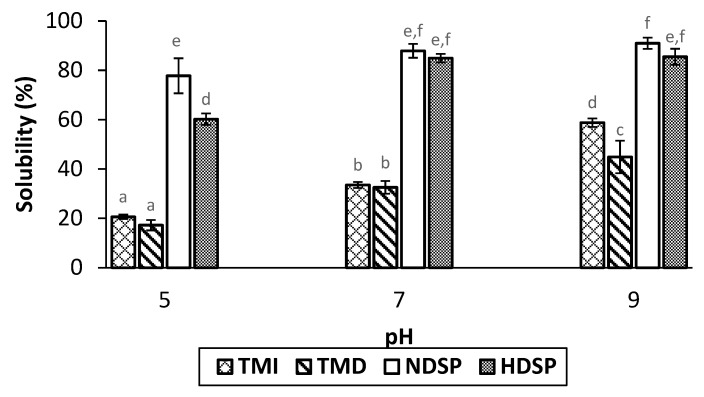
Protein solubility at pH 5, 7 and 9 of TMI, TMD, NDSP, and HDSP fractions. Different letters indicate significant differences (*p* < 0.05).

Figure 3 shows the protein solubility of TMI, TMD, NDSP, and HDSP fractions at pH 5, 7, and 9. Both the type of fraction and the pH had a significant impact on protein solubility (*p* < 0.0001). Similar solubilities were obtained for HDSP and NDSP at pH 7 and 9, whereas the solubility of HDSP was calculated to be lower than NDSP at pH 5. As expected, and whatever the pH value, protein fractions recovered from HDSP and NDSP (soluble fraction) were more soluble than TMI and TMD. A similar increase in solubility was observed for TMI and TMD when the pH increased, except at pH 9 where the solubility of TMI was higher than TMD. Globally, results showed that fractions had minimum and maximum solubilities at pH 5 and 9, respectively.

### 3.4. Foaming Properties

**Figure 4:**
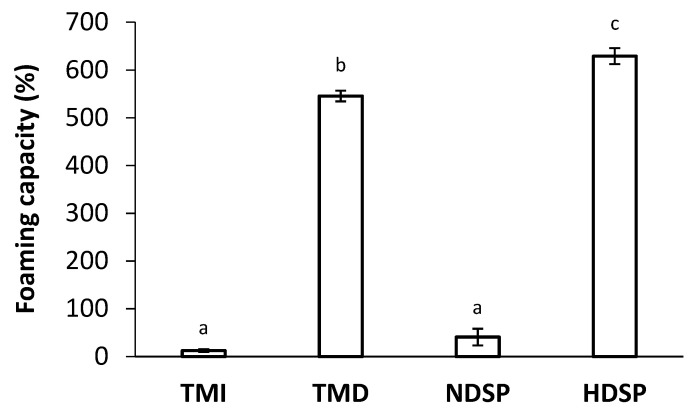
Foaming capacity of TMI, TMD, NDSP, and HDSP. Different letters indicate a significant difference (*p* < 0.05).

**Figure 5:**
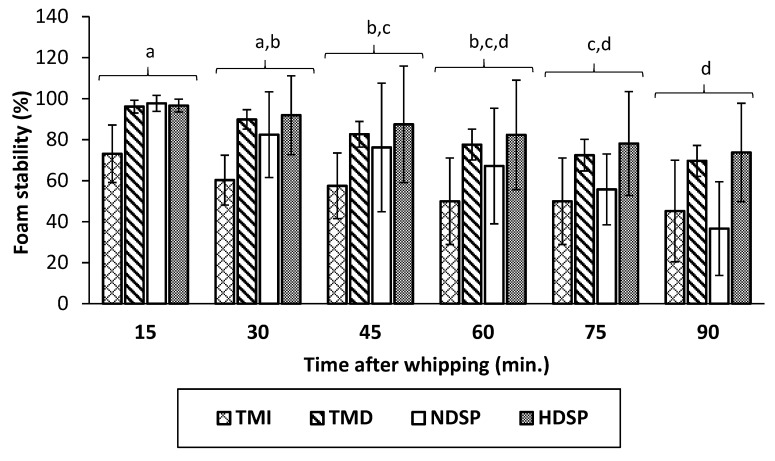
Foam stability values as a function of time after whipping of TMI, TMD, NDSP, and HDSP. Different letters indicate significant differences (*p* < 0.05).

Foaming capacity and foam stability of TMI, TMD, NDSP, and HDSP are presented in Figure 4 and Figure 5. Low foaming capacities of 13% and 41% were measured for TMI and NDSP, respectively. However, the foaming capacities for TMD and HDSP were drastically improved (546% and 629%, respectively), even if HDSP exhibited the highest foaming capacity. Foam stability values were similar for all insect fractions (*p* > 0.05) and decreased similarly as a function of time for both defatted and non-defatted fractions. More specifically, 90 min after whipping, a decrease to about 61% of initial foam volume was obtained.

## 4. Discussion

A growing number of studies are becoming available regarding the impact of processing methods on protein techno-functionalities [15]. However, no information is specifically available concerning the impact of the defatting step on mealworm protein profiles and functionalities. Consequently, the purpose of this study was to compare the protein profiles of hexane-defatted and non-hexane-defatted mealworm meals and protein extracts and to evaluate the impact of hexane on protein solubility and foaming capacity. Our results showed that defatting with hexane had little impact on protein profile. However, the 86 kDa early-staged encapsulation inducing protein and hexamerin 2 were more abundant in hexane-defatted protein extracts than in non-hexane-defatted protein extracts. Moreover, compared to non-defatted meals and protein extracts, solubility was reduced and foaming properties were largely improved for defatted fractions.

The initial fat content of 28.5% was within the normal range of similar studies [16,19,35] and residual lipids in hexane-defatted fractions did not exceed 0.4%, which is comparable to the results obtained by Choi et al. (2017) for defatted *T. molitor* meals [16]. The low-fat content of NDSP (0.5%) indicates that the cold centrifugation step following aqueous solubilization of proteins allows lipid to be extracted as efficiently as the hexane-defatting step by the formation of an easily removable solid fat layer. However, unlike the hexane-defatting step, cold centrifugation can only be performed after aqueous protein extraction, which resulted in a lower protein content in the soluble NDSP fraction compared to HDSP, since some lipid binding proteins might have been removed along the lipid fraction during the cold centrifugation step in the process to obtain the NDSP protein extract (Table 1).

Our results showed that the initial *T. molitor* (TMI) protein content of 38.6% was close to that of Purschke et al. (2019), who obtained 39% for dried *T. molitor* larvae [36]. As previously reported, hexane-defatting increased the protein content of TMI compared to TMD by removing lipids [19,37]. Our results also confirmed an increase in the protein content of soluble protein extracts of HDSP compared to NDSP (62.7% and 54.0%, respectively) after subsequent aqueous protein extraction. This result could be explained by the fact that protein-lipid interactions limit protein solubility during protein extraction [14,38].

Two-dimensional gel electrophoresis coupled to proteomic identification of the resulting spots was performed with all four fractions to understand the effects of the processing methods, especially the defatting step, on protein abundance and profile. Both proteomic and 2D electrophoresis results were consistent with previously published work on the characterization of *T. molitor* proteins. These studies identified many pan-allergens, such as muscle proteins (actin, myosin, tropomyosin) [15,32,39], hemolymph proteins (hexamerin 1 and 2) [15,32,39], and proteins associated with various metabolic activities (arginine kinase, α-amylase) [39,40,41], to be the most abundant in *T. molitor.* Overall, the protein profiles were quite similar for the four fractions although specific differences in the abundance of major proteins were observed. Interestingly, the biggest differences were observed between whole protein meals (TMI and TMD) and soluble protein extracts (NDSP and HDSP), rather than between hexane-defatted and non-hexane-defatted fractions. Indeed, five protein spots corresponding to the 12 kDa hemolymph protein (spot #4 and #5), α-amylase (spot #8), melanin-inhibiting protein (spot #10), and chitinase (spot #11) were predominantly abundant in extracted proteins fractions. These proteins, which are involved in different metabolic processes, are highly soluble in hemolymph and other aqueous solutions [42], which could explain the difference observed between the meal and extracted proteins fractions. Some differences in the protein profile were also detected between hexane-defatted and non-hexane-defatted soluble extracts. For example, actin-87E-like protein of *Tribolium castaneum* (spot #1), 86 kDa early-staged encapsulation inducing protein and Hexamerin 2 (spot #6) were present only in HDSP and not in NDSP. While the lack of knowledge about the structure and function of insect proteins make it difficult to formulate a hypothesis or draw any conclusion about the roles of these proteins [15], they are undoubtedly soluble. Furthermore, insect hexamerins, which have been studied in more depth, can provide some insight into the mechanisms that are active during the production of *T. molitor*. Indeed, hexamerins are synthesized and stored in the fat body of the insect in its larval form [43]. The fat body is a heterogenous organ consisting of cells containing multiple lipid droplets [44]. Consequently, their high abundance in HDSP fractions can be explained by the fact that those proteins are imprisoned in adipocyte cells and are only released during hexane-defatting, thus making them more abundant in HDSP. As many proteins are synthesized in the fat body, this hypothesis could also explain the abundance of 86 kDa early-staged encapsulation inducing protein in HDSP compared to NDSP [44]. Finally, it should be noted that the cockroach allergen-like protein was identified as the major constituent of both protein spots #2 and #3, whereas protein spot #3 in TMI had very low intensity. Indeed, some TMI proteins did not migrate on the gel during the second dimension, as can be observed by the presence of an intense band at 250 kDa (Figure 2A).

Proteins were probably unable to migrate in TMI fractions, since they were aggregated with other components of the cuticular matrix, such as chitin and lipids [45,46]. This phenomenon of protein aggregates was also observed by Boukil et al. (2020) for non-processed *T. molitor* meal [32].

The solubility of *T. molitor* proteins was minimal at pH 5 and increased at pH values of 7 and 9 (Figure 3). Our results were consistent with the previous work of Buβler et al. (2016) and Azagoh et al. (2016) who observed that the lowest protein solubility was obtained between pH 3 and 5, corresponding to the average pI. Indeed, at this pH value, the protein-water interactions are at their minimum, thereby inducing their insolubility. Interestingly, the same authors reported maximal edible insect protein solubility at alkaline pH values ranging from 7 to 12, depending on the fraction [37,47]. Borremans et al. (2020) observed that the solubility of hexane-defatted *T. molitor* meals (i.e., TMD) decreased compared to full-fat meals (i.e., TMI) at pH values above 8 [9,22], as obtained in our study (Figure 3). However, the same tendency was not repeatable for soluble protein extracts, since the pH increase did not affect the protein solubility of NDSP compared to HDSP. Interestingly, this phenomenon was observed at pH 5 since HDSP solubility was lower than that of NDSP. This could be related to a change in the average pI value due to the variation in protein molecular structure and protein content caused by the defatting process [48], evidenced by the profile differences observed in Figure 2. Overall, these results suggest that little protein denaturation occurred during the defatting process, as very few differences were observed, especially at neutral pH, between whole protein meals (TMI and TMD) and soluble protein extracts (NDSP and HDSP), respectively [49].

Our results also showed the highest foam capacity for hexane-defatted protein fractions TMD and HDSP. Yi et al. (2013), Zielińska et al. (2018), and Stone et al. (2019) reported that non-defatted *T. molitor* had poor foaming properties, which was confirmed by our results for TMI and NDSP (Figure 4) [7,8,24]. Borremans et al. (2020) evaluated the foaming properties of hexane-defatted *T. molitor* meal and showed increased foam capacity from 94% in the control sample to 540% for the defatted mealworms, which is similar to what is observed for TMD [22]. Lastly, foaming capacity of HDSP (629%) happened to be higher than the one of egg albumen (575%) which is commonly used as a foaming agent in food formulation. Akpossan et al. (2015) and Kim et al. (2020) also determined that the foaming capacity of defatted *Imbrasia oyemensis* meals and *Protaetia brevitarsis* protein extracts were superior to that of full-fat meals and non-defatted protein extracts, respectively [20,50]. Kim et al. (2020) partly attributed these results to the high-fat content of the non-defatted insect protein extracts, but that is not the case in this study, according to the proximate composition of NDSP, which still showed poor foaming capacity [20]. On the other hand, as experimented and discussed by Mishyna et al. (2019) for *Schistocerca gregaria* and *Apis mellifera*, higher foaming capacity could be attributed to the higher protein content of defatted fractions as well as the alteration of protein intrinsic molecular properties (e.g., partial protein unfolding) during the transformation process [9].

## 5. Conclusions

This study demonstrated that mealworm larvae processing methods, including defatting, modified the meal and protein extract’s protein profile and altered solubility and foaming capacity. Specifically, hexane-defatting of *T. molitor* meal before aqueous extraction of proteins increased the total protein content and abundance of the 86 kDa early-staged encapsulation inducing protein and hexamerin 2 of hexane-defatted protein extracts compared to the non-hexane-defatted extracts. Hexane-defatted *T. molitor* meals and protein extracts also had highly increased foaming capacity. Furthermore, *T. molitor* protein fractions exhibited the highest solubility at pH values above 7, which could facilitate their incorporation into various food systems. While further research on the effect of different processing steps (e.g., grinding, drying, protein extraction etc.) on insect protein quality and techno-functional properties is required to improve their use as a protein source in food formulations, this study provides insights on how hexane-defatted *T. molitor* protein ingredients could be incorporated into different foods, especially as foams, meringues, cakes, and mousses.

## Figures and Tables

**Table 1:** Proximate composition of *T. molitor* fractions at different processing stages expressed on a dry basis.

Processing Stage	Protein	Lipid	Chitin	Ash	Moisture	Total Solids
	% (*w*/*w*)					
TMI	38.6 ± 1.4 ^a^	28.5 ± 1.0 ^a^	5.0 ± 0.6 ^a^	3.4 ± 0.1 ^a^	9.7 ± 3.2 ^a^	90.3 ± 3.2 ^a^
TMD	59.1 ± 0.9 ^b,c^	0.4 ± 0.2 ^b^	7.8 ± 0.6 ^b^	4.8 ± 0.1 ^a^	4.8 ± 0.1 ^b^	95.2 ± 0.1 ^b^
HDSP	62.7 ± 3.0 ^c^	0.3 ± 0.1 ^b^	0.0 ± 0.0 ^c^	8.4 ± 1.1 ^b^	7.3 ± 0.8 ^a,b^	92.7 ± 0.8 ^a,b^
NDSP	54.0 ± 5.0 ^b^	0.5 ± 0.1 ^b^	0.0 ± 0.0 ^c^	10.3 ± 1.3 ^b^	9.3 ± 1.2 ^a,b^	90.7 ± 1.2 ^a,b^

Different letters in the same column indicate significant differences (*p* < 0.05).

**Table 2:** Proteins identified by proteomics in *T. molitor* 2D gels.

**#**	Identified Proteins	Molecular Weight MW (kDa)	UniProt ID	Number of Unique Peptides	Coverage (%)	Total Spectrum Count	Most Abundant in Fractions
**1**	Actin-87E-like protein (*Tribolium castaneum*)	42	D6WF19	39	77	736	TMI, TMD, and HDSP
**2**	Cockroach allergen-like protein (*Tenebrio molitor*)	65	Q7YZB8	5	11	53	TMI, TMD, NDSP, and HDSP
**3**				9	15	158	TMD, NDSP, and HDSP
**4**	12 kDa hemolymph protein b (*Tenebrio molitor*)	14	Q7YWD7	2	81	297	NDSP and HDSP
**5**				2	81	271	
**6**	86 kDa early-staged encapsulation inducing protein (*Tenebrio molitor*)	91	Q9Y1W5	21	27	93	HDSP
	Hexamerin 2 (*Tenebrio molitor*)	85	Q95PI7	12	20	81	
**7**	Arginine kinase (Fragment) (*Tenebrio molitor*)	27	A0A0U4ARJ8	8	85	414	TMI and TMD
**8**	α-amylase (*Tenebrio molitor*)	51	P56634	30	80	731	NDSP and HDSP
**9**	28 kDa desiccation stress protein (*Tenebrio molitor*)	25	Q27013	21	49	202	TMI, TMD, NDSP, and HDSP
**10**	Melanin-inhibiting protein (*Tenebrio molitor*)	40	Q4LE89	25	64	225	NDSP and HDSP
**11**	Chitinase (*Tenebrio molitor*)	40	Q7YZB9	11	38	156	NDSP and HDSP

## Data Availability

Data contained within the article are available from the authors.

## Sample Availability

Samples of the compounds are not available from the authors.
